# Regulation of Marginal Zone B-Cell Differentiation by MicroRNA-146a

**DOI:** 10.3389/fimmu.2016.00670

**Published:** 2017-01-16

**Authors:** Jennifer K. King, Nolan M. Ung, May H. Paing, Jorge R. Contreras, Michael O. Alberti, Thilini R. Fernando, Kelvin Zhang, Matteo Pellegrini, Dinesh S. Rao

**Affiliations:** ^1^Division of Rheumatology and STAR Program, Department of Medicine, University of California Los Angeles, Los Angeles, CA, USA; ^2^Cellular and Molecular Pathology Ph.D.Program, Department of Pathology and Laboratory Medicine, University of California, Los Angeles, CA, USA; ^3^Department of Pathology and Laboratory Medicine, University of California Los Angeles, Los Angeles, CA, USA; ^4^Department of Biological Chemistry, University of California Los Angeles, Los Angeles, CA, USA; ^5^Howard Hughes Medical Institute, University of California Los Angeles, Los Angeles, CA, USA; ^6^Jonsson Comprehensive Cancer Center, University of California Los Angeles, Los Angeles, CA, USA; ^7^Molecular Cell and Developmental Biology, University of California Los Angeles, Los Angeles, CA, USA; ^8^Eli and Edythe Broad Center of Regenerative Medicine and Stem Cell Research, University of California Los Angeles, Los Angeles, CA, USA

**Keywords:** B-cell development, marginal zone B-cells, microRNA, notch signaling, gene regulation

## Abstract

B-cell development in the bone marrow is followed by specification into functional subsets in the spleen, including marginal zone (MZ) B-cells. MZ B-cells are classically characterized by T-independent antigenic responses and require the elaboration of distinct gene expression programs for development. Given their role in gene regulation, it is not surprising that microRNAs are important factors in B-cell development. Recent work demonstrated that deficiency of the NFκB feedback regulator, miR-146a, led to a range of hematopoietic phenotypes, but B-cell phenotypes have not been extensively characterized. Here, we found that miR-146a-deficient mice demonstrate a reduction in MZ B-cells, likely from a developmental block. Utilizing high-throughput sequencing and comparative analysis of developmental stage-specific transcriptomes, we determined that MZ cell differentiation was impaired due to decreases in Notch2 signaling. Our studies reveal miR-146a-dependent B-cell phenotypes and highlight the complex role of miR-146a in the hematopoietic system.

## Introduction

Distinct subsets of immunoglobulin-expressing B-cells have been described in the spleen, including follicular (FO) B-cells, marginal zone (MZ) B-cells, and other, more rare, B-cell subsets. In general, the former subset is important for T-dependent B-cell responses to pathogens, while the latter is thought to play a role predominantly in T-independent B-cell responses and lipid antigen presentation to natural killer T cells (1). Both subsets are thought to derive from bone marrow-derived naïve B-cells that arrive in the spleen, where they can be recognized as immature transitional B-cells (T1, T2, and T3 B-cells). The development of MZ B-cells from these transitional subsets is thought to be regulated by several mechanisms, including (1) strength of B-cell receptor (BCR) signaling, (2) B-cell activating factor (BAFF)/receptor for BAFF (BAFF-R), (3) Notch2 signaling, (4) integrins and chemokines required for MZ retention in the spleen, and (5) NFκB signaling (2). BCR engagement combined with BAFF/BAFF-R signaling promotes B-cell survival and separately MZ B development (3). While both FO and MZ B-cells depend to some degree of BAFF/BAFF-R and NFκB signaling, the cell surface receptor Notch2 is critical specifically for MZ B-cell development. Activation by the ligand Delta-like-1 expressed by endothelial cells of red pulp venules catalyzes proteolytic cleavage of Notch2, releasing its intracellular domain, and resulting in nuclear translocation and downstream transcriptional effects (4).

It is likely that several other factors play roles in regulating the expression and activity of these key regulators of MZ B-cell development. MicroRNAs (miRNAs) are a family of small non-coding RNAs, 19–23 nt in length, which predominantly act *via* posttranscriptional repression of target messenger RNAs (mRNAs) by binding to the complimentary 3′-untranslated region (UTR) of the mRNA. To date, miRNAs have been implicated in a wide range of biologic processes, including hematopoietic cell development, immune function, autoimmunity, and oncogenesis (5). A single miRNA can target multiple mRNA transcripts and target mRNAs may be controlled by multiple miRNAs, thus adding a layer of complexity to cellular gene expression. Recent work has indicated the general importance of miRNAs in modulating the differentiation of splenic B-cell subsets. A B-cell specific knockout of Dicer, an endoribonuclease required for miRNA biosynthesis, resulted in a preferential development of MZ B-cells in mice (6). In addition to a general role for Dicer, specific miRNA loss or deregulation has been associated with various phenotypes within the B-cell compartment (7).

miR-146a is an NFκB-induced miRNA that shows high expression in spleen tissue, in particular splenic myeloid, T, and B-cells (8, 9). Studies using *Mir146a^−/−^* (KO) mice were found to have hyperactivated T FO helper cells and germinal centers (10), autoimmunity (8), T cell hyperactivation (11), and myeloid and lymphoid tumors (12) as a consequence of loss of feedback regulation *via* derepression of miR-146a targets, *Traf6, Irak1*, and *Stat1* (9, 13). Although these studies have well characterized miR-146a’s effects in myeloid and T cell subsets, the effects on B-cells are not well understood. In our study, we found that *Mir146a^−/−^* mice show an age-independent defect in MZ B-cell development. We have carefully characterized this defect, finding that KO mice show an increase in the preceding transitional B-cell stages and intact splenic retention, indicating a block in development. Using a combination of high-throughput sequencing, molecular biological and cellular-based approaches, we identified that this developmental block results from deregulation of the Notch2 pathway.

## Materials and Methods

### Mice

miR-146a-deficient (*Mir146a^−/−^*) mice were generated as described previously (8). Wild-type (WT) mice (C57B/6) and TCRαβ-deficient (*TCR*β^−/−^) mice (002118) were purchased from Jackson laboratories and kept under pathogen-free conditions at the University of California, Los Angeles. All mouse studies were approved by the UCLA Office of Animal Research Oversight.

### Flow Cytometry

Bone marrow, spleen, and blood were obtained from euthanized mice and red blood cell lysis buffer was used to lyse the single cell suspensions. For intracellular staining, cells were fixed in 2% paraformaldehyde at 37°C for 10 min and then blocked with Fc block for 20 min. Then, they were stained in 1× PBS with 10% FBS and 0.1% Triton X-100 for 20 min in the dark, and subsequently washed with 1× PBS with 4% FBS. Fluorochrome-conjugated antibodies and subset definitions are listed in Tables S1 and S2 in Supplementary Material. Initial spleen gating strategy is shown in Figure S1A in Supplementary Material. Flow cytometry was performed on an LSRII and data were analyzed using FlowJo software.

### Sorting of Splenic B-Cell Subsets

Spleens pooled from three to five mice were stained according to FACS surface markers as noted in Table S2 in Supplementary Material. Cells were sorted into T1, T2, T3, FO B-cells, and MZ B-cells *via* FACS Aria.

### RNA Sequencing (RNA-Seq) and Analysis

Total RNA was extracted from WT and KO B-cell subsets using Qiazol using the Qiagen miRNEasy mini kit with additional on column DNAse I digestion. Following isolation of RNA, cDNA libraries were built using the Illumina TruSeq RNA Sample Preparation kit V2 (RS-122-2001). An Agilent Bioanalyzer was used to determine RNA quality (RIN >8) prior to sequencing. RNA-Seq libraries were sequenced at the Broad Stem Cell Research Center sequencing core (UCLA). Libraries were sequenced on an Illumina HiSeq 2000 (single-end 100bp). Raw sequence files were obtained using Illumina’s proprietary software and are available at NCBI’s Gene Expression Omnibus (Accession GSE93252). We first filtered out reads with low quality and reads containing sequencing adapters and then mapped raw reads to the mouse reference genome (UCSC mm10) with the gapped aligner Tophat allowing up to two mismatches. We supplied the UCSC mm10 gene model to Tophat as the reference genome annotation. Only reads uniquely aligned were collected. In total for all libraries sequenced, 365,022,996 reads were uniquely mapped (corresponding to an overall mappability of 91.7%) and used for further analysis. Transcript expression levels were quantified using RPKM units (Reads Per Kilobase of exon per Million reads mapped) using customized scripts written in Perl. Differential expression analysis was performed using both DESeq and edgeR in R (http://www.R-project.org). Raw read counts were used and modeled based on a negative binomial distribution. The multiple testing errors were corrected by the false discovery rate (FDR). We considered genes as differentially expressed if (1) the FDR was less than 0.05, (2) the expression ratio between two time points was >2×, (3) the maximal RPKM value for at least one group in the comparison was >1, and (4) there was agreement between DESeq and edgeR. These differentially expressed genes were then examined from the T2 to MZ and T2 to FO stages in both WT and KO cells. We then focused on genes only found in the T2 to MZ transition in WT and compared them to those in the T2 to MZ transition in KO B-cells. The *Z*-scores for each of these unique gene subsets were calculated, scaled, centered, and displayed as a heat map.

### RT-qPCR

RNA was collected from corresponding B-cell samples and reverse transcribed using the qScript reagent (Quanta Biosciences). RT-qPCR was performed with the StepOne Plus Real-Time PCR System (Applied Biosystems) using PerfeCTa SYBR Green FastMix reagent (Quanta Biosciences) or TaqMan MicroRNA Assay (Life Technologies). Primer sequences used are listed in Table S2 in Supplementary Material.

### Western Blot

B220^+^ cells were isolated from WT and KO mice using MACS, treated with LPS, and cultured for 72 h. B-cells were then lysed with RIPA buffer (Boston BioProducts) and Halt Protease and Phosphatase Inhibitor Cocktail (Thermo Scientific). Equal amounts of protein lysate [quantification by bicinchoninic acid protein assay, BCA (Thermo Scientific)] were separated using electrophoresis on a 10% SDS–PAGE and blotted on a nitrocellulose membrane. Numb Rabbit monoclonal (C29G11)(#2756) and β Tubulin Rabbit polyclonal (#2146) antibodies were used (Cell Signaling Technologies). HRP-conjugated secondary antibodies were from Santa Cruz Biotechnology.

### Luciferase Reporter Assay

The entire *Numb* 3′-UTR (1,978−3,382 nt; GenBank ID: NM_001136075) containing the miR-146a site was cloned into the pmiRGlo dual luciferase vector (Promega). The miR-146a seed sequence AGTTCTCA (2,596−2,603 nt) was mutated to CTCATAGT and also cloned into pmiRGlo. A similar strategy was used for cloning a 2 kb segment of the *Notch2* 3′-UTR (7,584−9,592 nt; GenBank ID: NM_010928) immediately downstream of the stop codon. The putative miR-146a seed sequence GTTCTCA (8,815−8,821 nt) was mutated to CAGTCTT and also cloned into pmiRGlo. Standard PCR and cloning methods were employed. TargetScan was used to predict miR-146a seed sequences. The *Traf6* 3′-UTR was cloned as previously described (14). HEK-293T cells were co-transfected with luciferase reporter vectors, with or without miR-146a expression vector using BioT transfection reagent (Bioland Scientific) as per the manufacturer’s instructions. Cells were lysed after 48 h, substrate was added, and luminescence was measured on a Glomax-Multi Jr (Promega).

### Statistical Analyses

Figures are graphed as mean with the SD of the mean for continuous numerical data. Dichotomized or ordinal-type histopathologic data are presented using bar graphs. Data were analyzed with two-tailed Student’s *t*-test, conducted using GraphPad Prism software, applied to each experiment as described in the figure legends. *p < 0.05, **p < 0.01, ***p < 0.001, and ****p < 0.0001.

## Results

### Deficiency of miR-146a Results in Decreased MZ B-Cells in the Spleen

**Figure 1:**
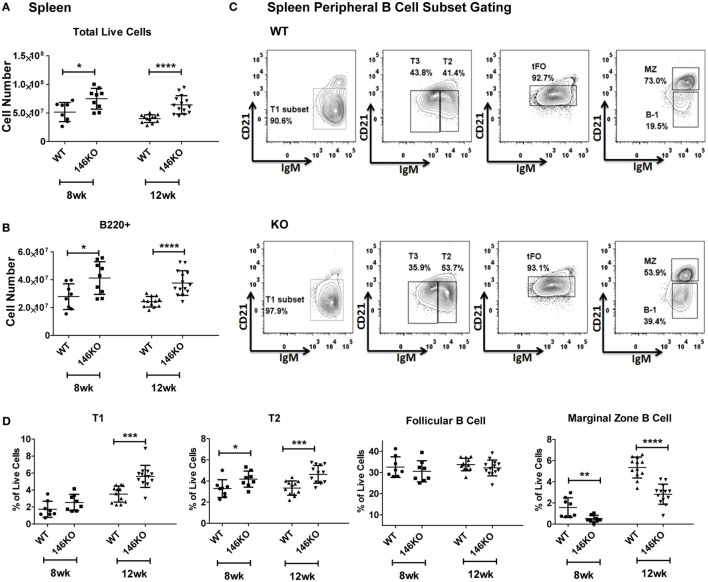
**Mir146a^−/−^ mice exhibit an increase in total splenic B-cells and immature splenic subsets, except exhibit a defect in marginal zone (MZ) B-cells**. Cell numbers of **(A)** total live cells (*p = 0.015, ****p < 0.0001) and **(B)** B220+ B-cells (*p = 0.021, ****p < 0.0001) are shown in young mice, ages 8 weeks (*n* = 8–9 mice/group) and 12 weeks (*n* = 12–14 mice/group). Values are mean ± SD. **(C)** Representative FACS plots show maturing B-cell subsets in spleens of wild-type (WT) mice and Mir146a*^−/−^* knockout (KO) mice at 12 weeks. **(D)** Analysis of spleen subsets from WT mice and KO mice are shown at early ages (*n* = 12–14/group). Increases in the transitional T1 (***p = 0.0001) and T2 (*p = 0.043, ***p = 0.0003) B cells are shown. MZ B-cells are decreased in KO at ages 8 weeks (**p = 0.008), 12 weeks (****p < 0.0001). Values are mean ± SD.

Previous studies have shown that *Mir146a^−/−^* mice show age-dependent changes in splenic cellularity and activated T cell phenotypes, starting from 2 to 4 months (10, 11). This is followed by autoimmune inflammatory disease, splenomegaly, lymphadenopathy, and myeloproliferation, and at very advanced ages by splenic myeloid sarcomas, lymphomas, and bone marrow failure (12). Given the high expression of miR-146a in B-cells and features of autoimmune disease in KO mice, we examined splenic B-cell subsets to better characterize miR-146a’s effect on B-cell maturation. Beginning with young mice, we confirmed that increases in total splenic cellularity (Figure 1A) and B220+ B-cells (Figure 1B) were apparent as early as 8–12 weeks old. We characterized immature transitional B-cells (T1, T2, and T3), FO cells, MZ B-cells, and B1 cells and compared them between WT and KO mice (Figure 1C) (15). Increased percentages and numbers in precursor T1, T2 (Figure 1D; Figure S2 in Supplementary Material), T3, and B1 cells (Figures S1C,D and S2 in Supplementary Material) were seen starting from 8 to 12 weeks in KO compared to WT. In addition, the overall number of FO B-cells started to trend higher in KO mice at 8 weeks of age and statistically significant differences were observed at 12 weeks of age in KO mice (Figure S2 in Supplementary Material). In contrast, MZ B-cells showed a significant decrease as early as 8 weeks of age (Figure 1D). This defect persisted until at least 18 weeks of age in the KO mice (Figure S3 in Supplementary Material), after which expansion of the myeloid compartment began to take over in KO spleens (12). In addition, an alternative gating analysis (Figure S1B in Supplementary Material) (1) showed that this defect was also present in MZ precursor (MZP) cells, again confirming a deficiency in the MZ development pathway (Figure S1E in Supplementary Material). Together, these results indicate a specific defect in the MZ subset of splenic B-cells, with an increase in T1 and T2 transitional zone B-cells, suggestive of defective differentiation into MZ B-cells in KO mice.

### Decreased MZ B-Cells in miR-146a-deficient Mice Are Due to Spleen-Intrinsic Defect and Not Bone Marrow Development Failure or Lack of Splenic Retention

**Figure 2:**
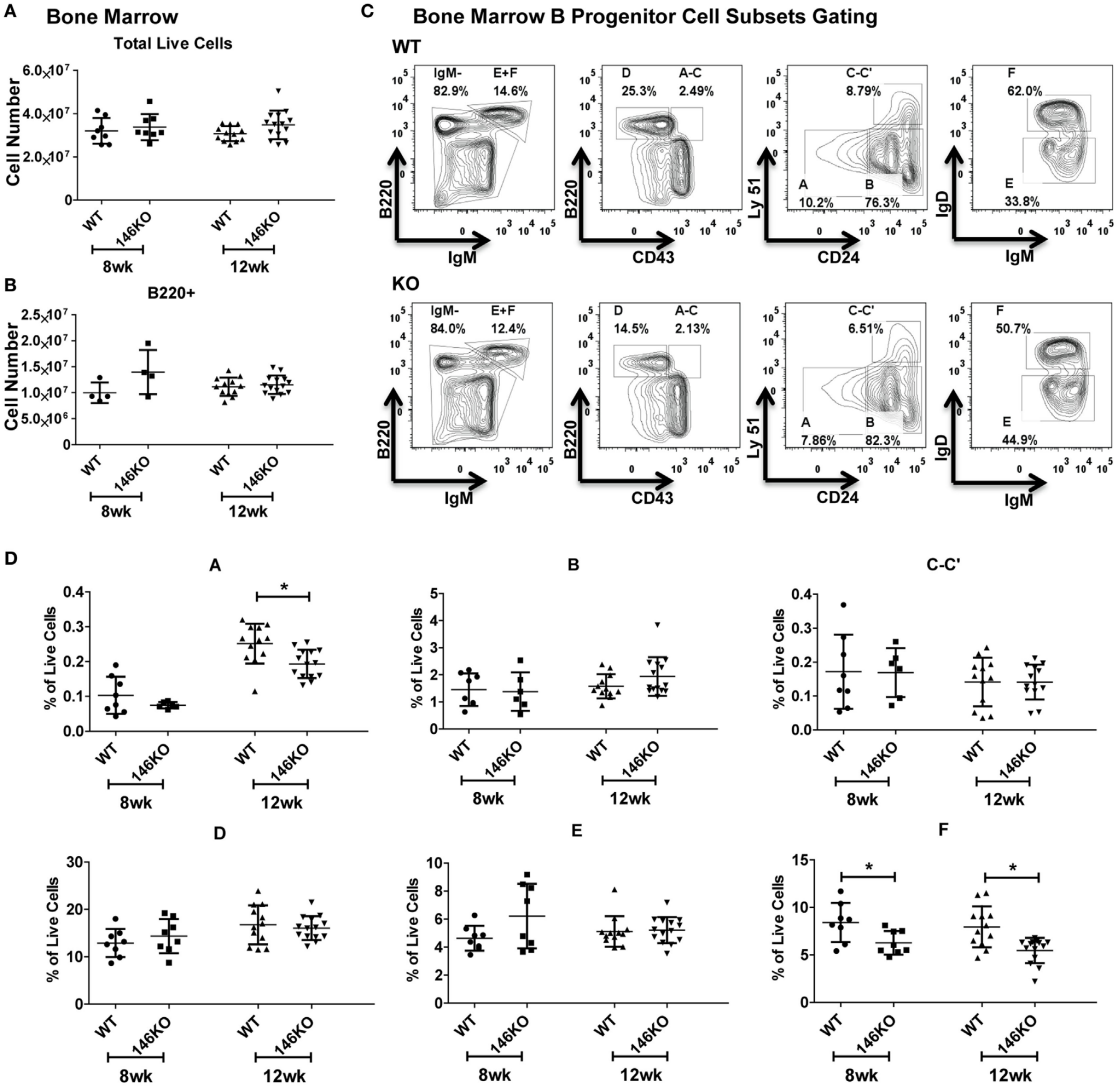
**Mir146a^−/−^ mice show intact bone marrow B-cell development**. Cell numbers of **(A)** total live cells (*p* = nss) and **(B)** B220+ B-cells (*p* = nss) are shown in young mice, ages 8 weeks (*n* = 8 mice/group) and 12 weeks (*n* = 12–14 mice/group), respectively. Values are mean ± SD. **(C)** Representative FACS plots show bone marrow B progenitors cells (Hardy Fractions) in bone marrow of WT (top panel) and KO mice (bottom panel) at 12 weeks. **(D)** Analysis of B progenitor cell subsets from WT and KO mice at early ages (8 and 12 weeks). Fraction A (*p = 0.006), fraction F at 8 weeks (*p = 0.025) and 12 weeks (*p = 0.001). Values are mean ± SD.

To fully characterize the origin of differences in splenic B-cell subsets between WT and KO mice, we looked upstream into the bone marrow to assess deficiencies in B-cell development. At the early ages of 8 and 12 weeks, there were no statistically significant differences in total live cells (Figure 2A) or B220+ B-cells (Figure 2B). We subsequently characterized bone marrow B progenitor cells (15) in WT and KO mice (Figure 2C). Fractions A–E in young mice remained similar, although at 12 weeks fraction A showed a decrease in KO mice (Figure 2D), which may represent the beginning of myeloproliferative disease or faster flux through this particular stage of development (12, 16). Hardy Fraction F cells, which represent mature bone marrow B-cells as well as recirculating cells, showed a decrease in KO mice, likely representing a decreased recirculating fraction secondary to increased retention of overall B-cells in the spleen. To ensure that this decrease in pre–pro B-cells was not due to decreases in hematopoietic stem cell/progenitor development, we examined hematopoietic stem cells (HSC), multipotent progenitors (MPP), lymphoid-primed multipotent progenitors (LMPP), early lymphoid (ELP), and common lymphoid (CLP) progenitors, and found no difference in young mice (Figures S4A,B in Supplementary Material), similar to prior work (16).

**Figure 3:**
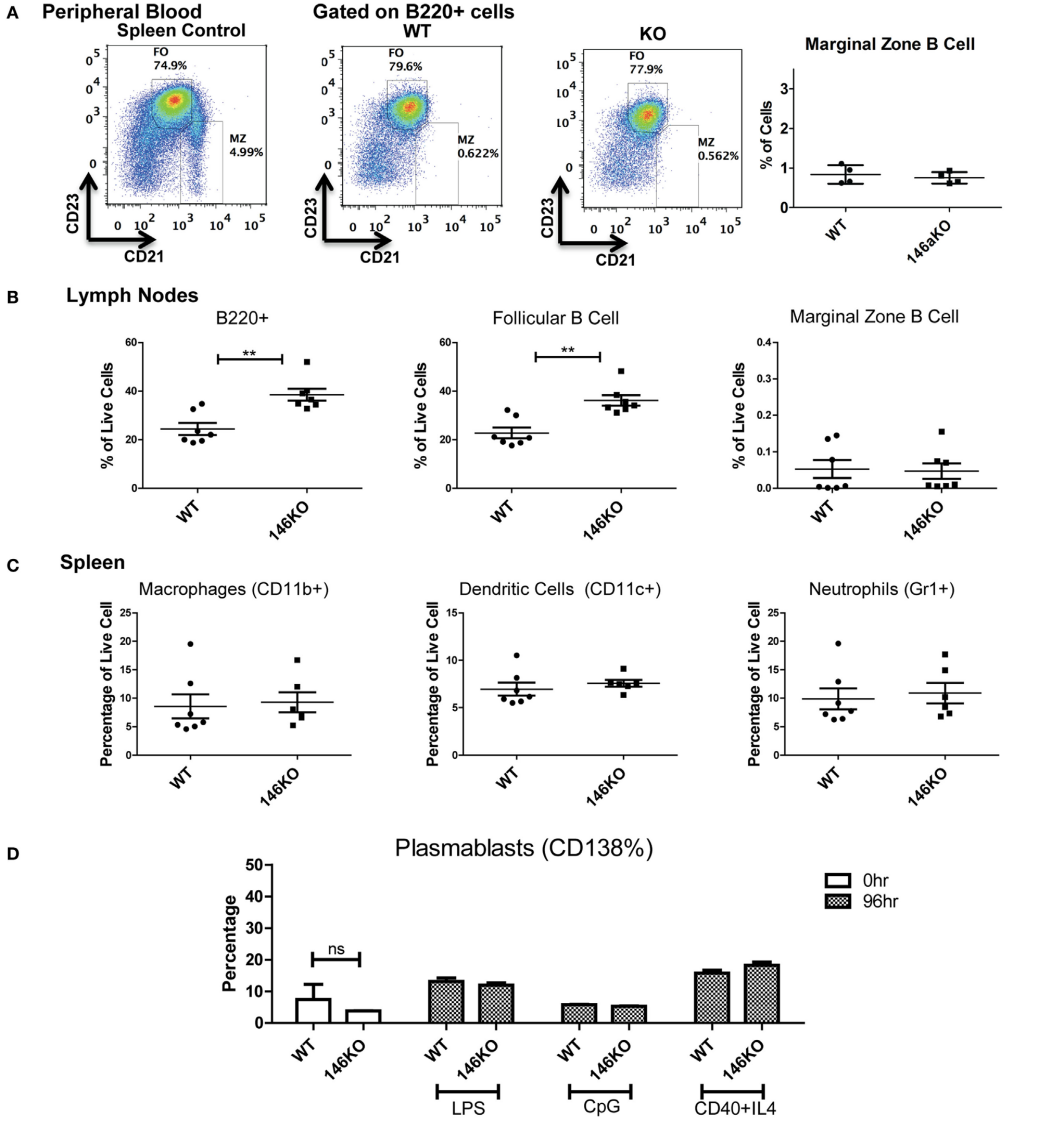

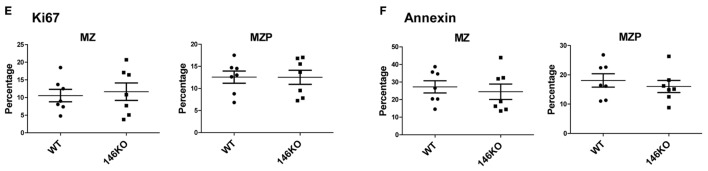
**Lower Mir146a^−/−^ marginal zone (MZ) B-cells are likely due to a spleen intrinsic developmental block**. **(A)** Peripheral blood B220+ cells show no statistically significant difference in circulating MZ or follicular cells (*n* = 4 mice/group). **(B)** Mature B-cell subsets are shown in peripheral lymph nodes (B220+ **p = 0.002; FO **p = 0.001) (*n* = 7 mice/group). **(C)** Splenic macrophages, dendritic cells, and neutrophils show no differences in WT vs. KO (*n* = 7 mice/group). **(D)** CD138+ plasmablasts are shown in unstimulated spleens (0 h) and 96 h poststimulation with CD40 + IL4, with no significant differences (repeated in duplicate). **(E)** Intracellular Ki67 and **(F)** Annexin V staining are shown for MZ and MZ precursor (MZP) cells (*n* = 7 mice/group).

In addition, we explored the possibility that a defect in MZ B-cells in KO mice may have been due to a lack of retention of these cells in the splenic niche. However, examination of the peripheral blood (17) showed no difference in MZ B-cells between WT and KO mice (Figure 3A). Also, there were no differences in MZ B-cells in lymph nodes (Figure 3B). However, overall B220+ cells and FO cells were increased in KO vs. WT, as consistent with spleen findings. Furthermore, because it is known that MZ cells may differentiate into plasmablasts after interacting with antigens presented from macrophages, dendritic cells, or neutrophils, we examined this possibility. It is known that myeloid lineage cells in the spleen of KO mice are significantly increased in older KO mice (8, 12, 16). Hence, we performed FACS analysis for macrophages, dendritic cells, and neutrophils in young 12-week-old mice and found that they were similar in WT and KO at this early age (Figure 3C). Furthermore, plasmablasts were then assessed in WT and KO spleens, and also following stimulation with LPS, CpG, or CD40 + IL4, and were not statistically different (Figure 3D). Together with a lack of significant changes in bone marrow development, these findings suggest a local spleen intrinsic defect leading to decreased MZ B-cell development.

Next, we explored whether the decrease in MZ B-cells was related to a developmental defect or due to an altered proliferation and/or apoptosis of MZ B-cells. Using intracellular Ki67 staining in conjunction with staining for B-cell developmental subsets, we determined that the proportion of Ki67 positive cells was statistically unchanged between WT and KO MZP and MZ B-cells (Figure 3E). Similarly, Annexin V staining revealed no statistically significant differences in apoptosis of these B-cell subsets (Figure 3F). In summary, these data strongly support the idea that the defect in MZ B-cells in *Mir146a^−/−^* mice is due to a developmental block in maturation of T2 cells into MZ B-cells.

### miR-146a-Deficient B-Cells Exhibit Altered Gene Expression during Maturation from Transitional to MZ Stages

**Figure 4:**
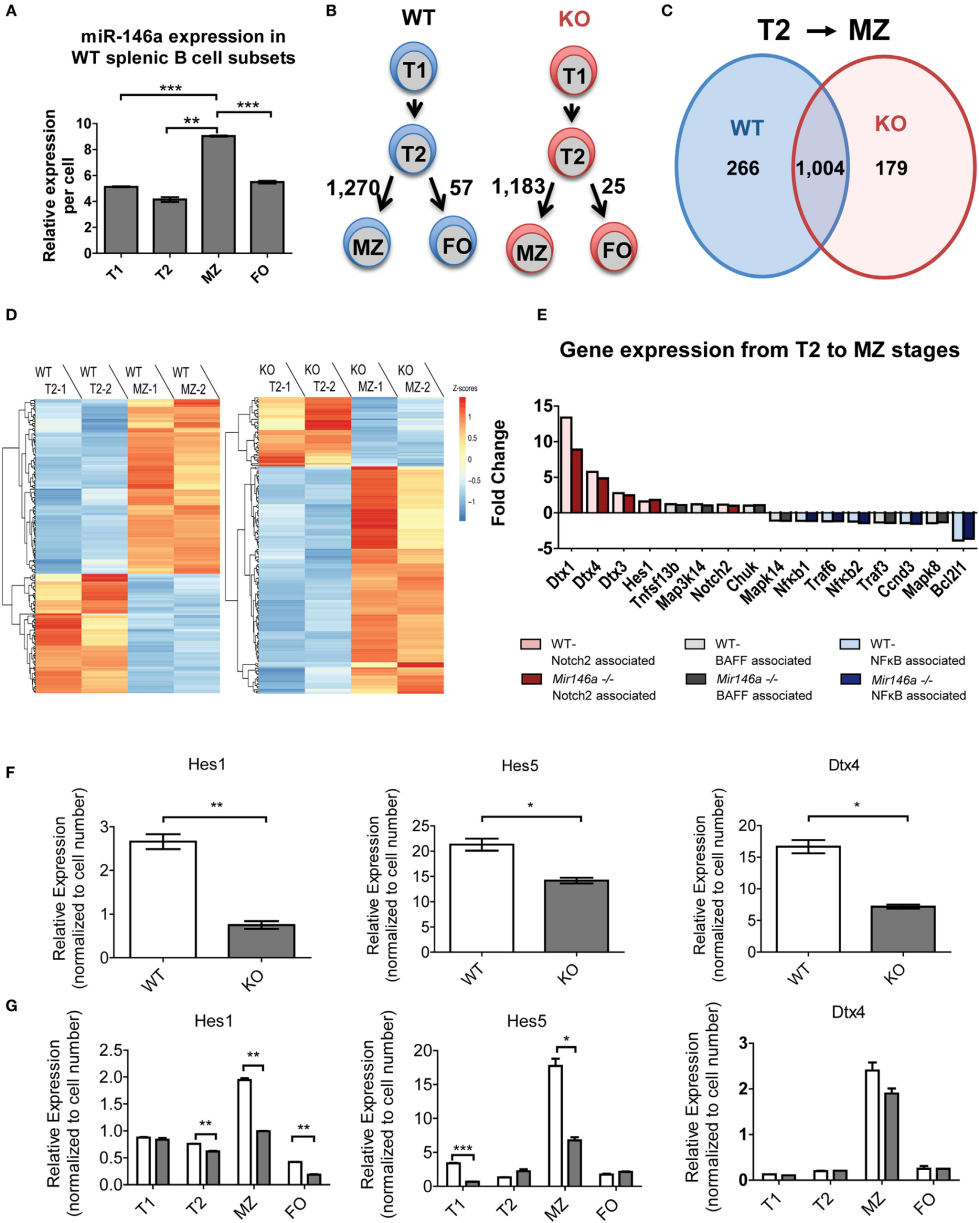
**RNA sequencing (RNA-Seq) reveals differentially expressed miR-146a-dependent genes at the T2 to MZ transition**. **(A)** Representative RT-qPCR showing relative expression levels of miR-146a in WT T1, T2, MZ, and FO splenic B-cell subsets per cell. (T1-MZ ***p = 0.0002; T2-MZ **p = 0.0015; MZ-FO ***p = 0.0009; repeated in duplicate). Values are mean ± SEM. **(B)** Schematic diagram depicting the number of differentially expressed genes between the T2 to MZ and T2 to FO transitions in both WT and KO mice. **(C)** Venn diagram comparing and contrasting the number of genes unique to and shared between the T2 to MZ transition in WT and KO mice. **(D)** Heat maps from the RNA-Seq data showing differentially expressed genes unique to the T2 to MZ transition in WT vs. genes unique to the T2 to MZ transition in KO (−1 and −2 denote replicates). **(E)** A waterfall plot of the RNA-Seq data displaying notable genes in three signaling pathways influencing MZ B-cell development. **(F)** Representative RT-qPCR analysis of Notch2-associated genes in bulk splenic B-cells 72 h after LPS stimulation repeated in duplicate (*Hes1* **p = 0.0099; *Hes5* *p = 0.032; *Dtx4* *p = 0.013). **(G)** Quantified relative expression of Notch2-associated genes in splenic B-cell subsets using RT-qPCR (Hes1 T2 **p = 0.0082, MZ **p = 0.0014, FO **p = 0.0019; Hes5 T1 ***p = 0.001, MZ *p = 0.011; repeated in duplicate).

To dissect the mechanistic basis of the observed MZ B-cell block in development, we undertook gene expression analyses of the splenic B-cell subsets. First, we examined whether miR-146a was differentially expressed in different splenic B-cells. Indeed, RT-qPCR revealed that MZ B-cells had the highest levels of miR-146a expression, with expression noted in all subsets (Figure 4A). Next, we performed RNA-Seq on WT and KO transitional (T1 and T2), MZ and FO B-cell subsets with biological replicates. Surprisingly, when we directly compared the corresponding B-cell stages between the WT and KO mice, only three to six genes showed differential expression. Of this limited group of differentially expressed genes, none were predicted miR-146a targets. The limited numbers of differentially expressed genes using direct comparison of subsets led us to consider alternate approaches. Given that there are very significant changes occurring in gene expression as a T2 cell commits to a MZ fate, we hypothesized that miR-146a alters the set of genes that are changing during this cell fate commitment. To this end, we utilized an alternative analysis comparing transitions between developmental stages of immune cells, similar to an approach that has been previously described (18). Interestingly, the transition from T2 to FO B-cells showed only 57 or 25 differentially expressed genes in the WT and KO, respectively (Figure 4B). In contrast, the T2 to MZ transition showed 1,270 or 1,183 differentially expressed genes in WT and KO, respectively (Figure 4B), indicating a large change in gene expression needed for commitment to the MZ over the FO fate. Focusing on the T2 to MZ transition, we compared the differentially expressed genes in WT and KO transcriptomes (Figure 4C). WT and KO mice showed 1,004 differentially expressed genes in common during the T2 to MZ transition; in addition, there were 266 differentially expressed genes unique to WT mice and 179 unique to KO mice. These unique gene subsets formed two clusters that either increased or decreased in expression during the T2 to MZ transition (Figure 4D). Interestingly, the subset unique to the KO mice showed the majority of genes increasing, although these genes as a class were not enriched for predicted miR-146a targets (data not shown).

### *Notch2* Activity Is Diminished in miR-146a-deficient MZ B-Cells

To further examine what genes may be changing, we analyzed transcriptional targets of pathways known to play a role in MZ B-cell differentiation, namely, *Notch2, BAFF*, and *NF*κ*B* (Figure 4E). Several known transcriptional targets of the *Notch2* pathway showed lesser fold change of reads per kilobase of transcript per million mapped reads (RPKMs) between T2 and MZ cells in KO compared to WT mice (Figure 3E, red). On the other hand, *BAFF*-associated genes (gray) and *NF*κ*B-*associated genes (blue) did not show a consistent change toward reduced or increased fold change at the T2–MZ transition between WT and KO. Because the RNA-Seq data demonstrated decreased expression of *Notch2* targets in KO MZ B-cells, we further validated these findings by examining *Notch2* transcriptional targets by RT-qPCR. First, we used bacterial lipopolysaccharide (LPS) to strongly induce miR-146a expression (9, 16) and screened for *Notch2* transcription targets using RT-qPCR at 72 h poststimulation. RT-qPCR revealed that KO B-cells showed significantly decreased expression of *Notch2* transcriptional targets *Hes1, Hes5*, and *Dtx4* (Figure 4F) (19, 20). We then examined expression of these candidate genes in primary splenic B-cell subsets, revealing reductions of *Hes1* and *Hes5* and a trend toward reduced *Dtx4*, specifically in MZ B-cells lacking miR-146a (Figure 4G). Together, these data suggest that KO mice have an altered gene expression profile during the T2–MZ transition, particularly downstream of *Notch2*, corresponding with the increased expression of miR-146a in the WT MZ population.

**Figure 5:**
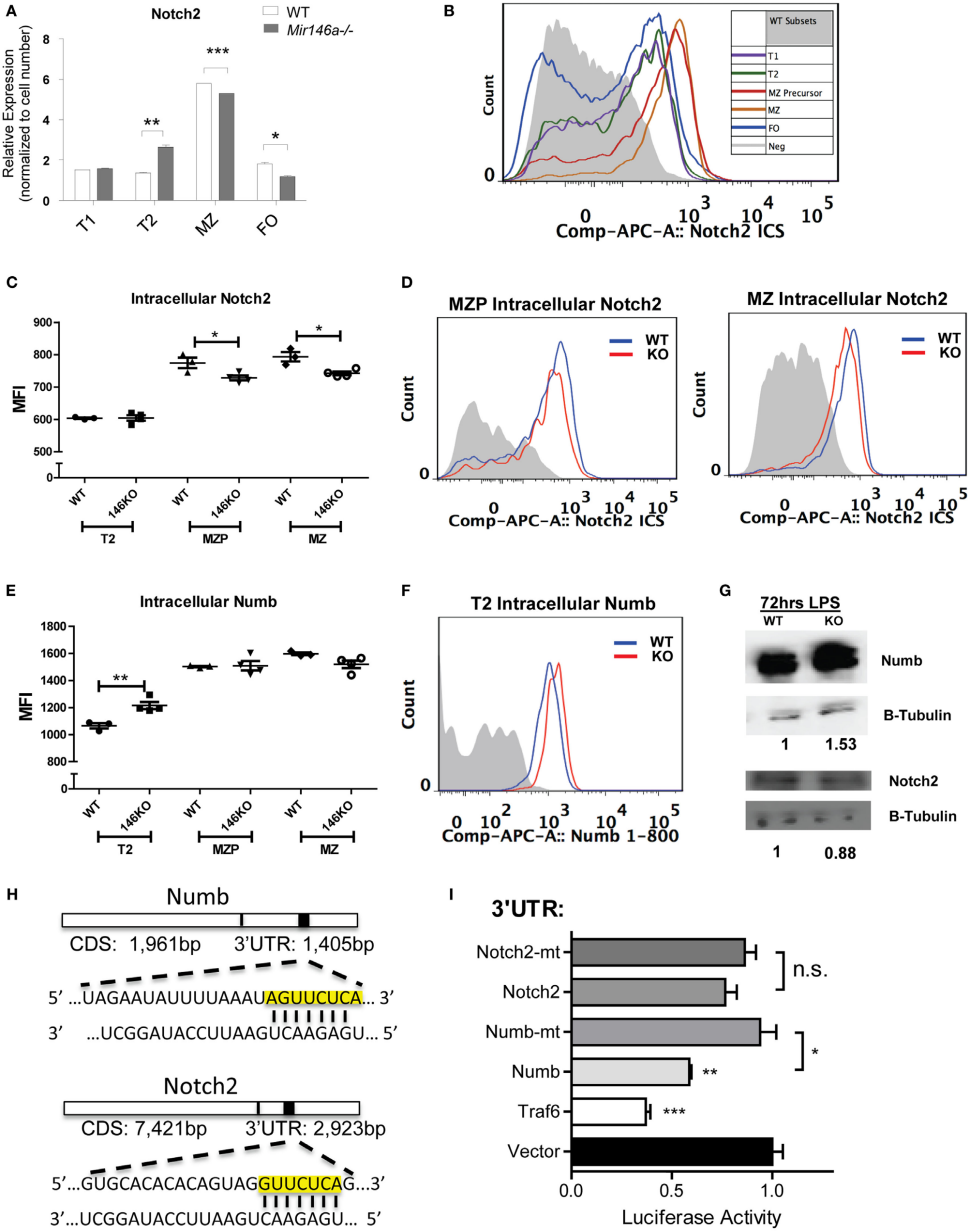
**Mir146a^−/−^ B-cells show decreased Notch2 and increased Numb, a direct target of miR-146a**. **(A)** Representative RT-qPCR quantification of Notch2 expression in splenic B-cell subsets in wild-type (WT) vs. KO (T2 **p = 0.0057, marginal zone (MZ) ***p = 0.0002, FO *p = 0.016; repeated in duplicate). **(B)** Representative FACS overlay of intracellular Notch2 expression in WT B-cell subsets. **(C)** Quantitation of median fluorescence intensity (MFI) of intracellular staining of Notch2 in T2, MZ precursors (MZP), and MZ B-cells (MZP *p = 0.035; MZ *p = 0.014; *n* = 3–4 mice/group, repeated in duplicate). **(D)** Representative overlapping histograms of Notch2 MZ subsets in WT vs. KO mice. **(E)** Quantitation of MFI of intracellular staining of Numb in T2, MZP, and MZ B-cells (T2 **p = 0.0083; *n* = 4–5 mice/group). **(F)** Representative overlapping histogram of T2 Numb in WT vs. KO. **(G)** Western blot shows increased Numb protein and decreased Notch2 protein in LPS-stimulated splenic B-cells from WT and KO mice. β-Tubulin was used as a loading control and fold change was calculated using ImageJ (repeated in duplicate). **(H)** *Numb* 3′-untranslated region (UTR) and *Notch2* 3′-UTR containing the putative seed sequences of miR-146a (yellow box) are shown. **(I)** Luciferase assays quantitating repression with MGP/miR-146a relative to MGP alone for each of the UTRs depicted. Each measurement is representative of firefly luciferase normalized to renilla luciferase and was performed in triplicate, with the experiment repeated at least three times (*Traf6* vs. Vector, ***p = 0.0004; *Numb* vs. Vector, **p = 0.0016; *Numb* vs. mutated *Numb*, *p = 0.012; *Notch2* vs. mutated *Notch2*). All values are mean ± SEM.

Interestingly, *Notch2* itself showed a mild but significant reduction at the mRNA level in KO MZ B-cells (Figure 5A). To characterize the effect of miR-146a deficiency on Notch2 protein, we utilized flow cytometry analysis. Notch2 is initially expressed as a surface receptor, and is internalized, cleaved, and endocytosed upon activation. Cell-surface expression of the transmembrane Notch2 receptor was not altered in the absence of miR-146a (Figure S5 in Supplementary Material). Subsequently, we examined intracellular expression of Notch2 in WT mice, noting that the highest expression of intracellular Notch2 was found in MZ and MZ precursor cells (Figure 5B). In line with a downstream effect on Notch2-mediated transcription, intracellular staining for Notch2 showed a significant decrease in KO MZ and MZP B-cells (Figures 5C,D). We then confirmed these findings *via* Western Blot. To obtain enough protein for analysis, we used LPS to stimulate the WT and KO bulk B-cells and found Notch2 protein was decreased in KO compared to WT cells at 72 h poststimulation (Figure 5G).

A similar analysis for NFκB transcriptional targets (Figures S6A,B in Supplementary Material) and for two known miR-146a targets, *Traf6* and *Irak1* (Figures S6C–F in Supplementary Material) (8, 9) failed to show statistically significant differences. In addition, a gene set enrichment analysis (GSEA) of the T2 to MZ differentially expressed genes in KO did not show enrichment in NFκB target genes (data not shown). Together, these data demonstrate that the decrease in MZ B-cells is most likely due to the reduction in *Notch2* activity and that miR-146a regulates the levels of Notch2.

### Numb, a Regulator of the Notch2 Pathway, Is a Direct Target of miR-146a

Previous studies have indicated that the adaptor protein Numb, a negative regulator of Notch, contains a predicted miR-146a-binding site within its 3′-UTR, but direct targeting has been observed by some groups, but not others (21, 22). One of the canonical functions of Numb is to inhibit Notch signaling by recruiting ubiquitin ligases to degrade cell surface-associated and intracellular Notch proteins (23). Numb may also directly bind to the notch intracellular domain (NICD) in the cytoplasm, thus preventing translocation into the nucleus and subsequent downstream transcription of *Hes1, Hes5*, and *Hey1* (19, 24). Here, we hypothesized that if Numb is derepressed in KO MZ B-cells, it may lead to degradation of Notch2 and subsequent inhibition of the gene expression program that is required for MZ B-cell development. Using intracellular FACS staining, we found that Numb was more highly expressed in KO transitional T2 cells (Figures 5E,F). Hence, this increase in Numb expression in the T2 subset developmentally precedes the decrease in *Notch2* expression in MZP and MZ B-cells, but the reciprocal relationship was not observed in the same cell types (Figures 5C,D). We then confirmed that Numb protein was increased by Western Blot in KO LPS-stimulated B-cells, consistent with a lack of repression by miR-146a (Figure 5G).

As mentioned above, the *Numb* 3′-UTR contains predicted miR-146a binding sites (Figure 5H). To assess direct repression of *Numb* by miR-146a, we cloned a ~1,300-bp fragment of the *Numb* 3′-UTR into the pmiRGlo luciferase reporter. Repression of the luciferase reporter was observed with co-transfection of miR-146a, and this repression was abolished upon mutation of the miR-146a seed sequence (see Materials and Methods). Of note, although *Notch2* also contained a predicted miR-146a-binding site within its 3′-UTR, luciferase activity was not repressed (Figures 5H,I). Together, our findings indicate that *Numb* is a direct target of miR-146a. Downstream, there is inhibition of Notch2 and the gene expression program required for MZ B-cell development. The relationship between miR-146a-mediated inhibition of Numb and Notch2 regulation by Numb requires further study.

### miR-146a Regulation of MZ B-Cell Development Is T-Cell Independent

**Figure 6:**
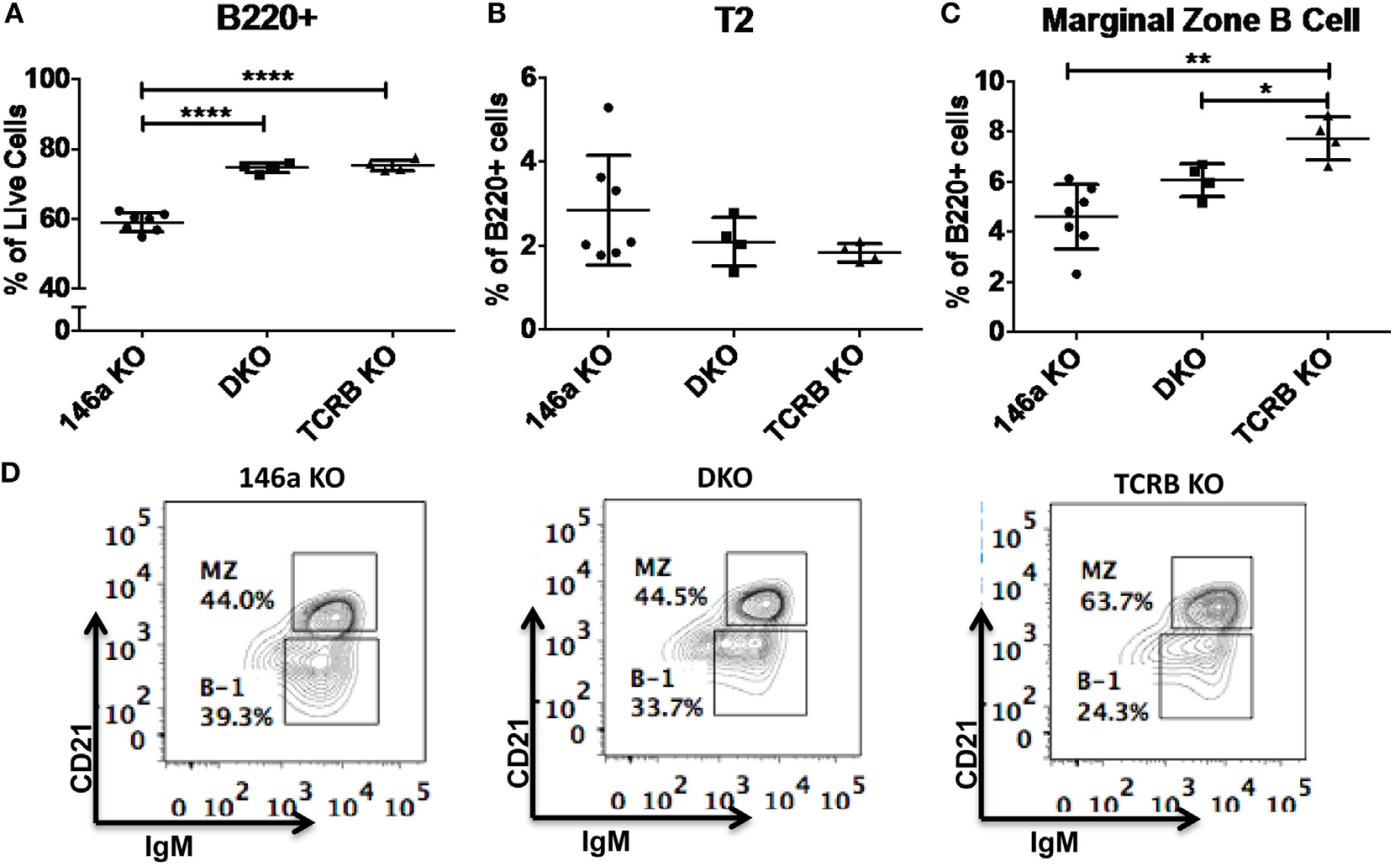
**Deficiency of marginal zone (MZ) B-cells in Mir146a^−/−^ mice is not T cell dependent**. Comparison of percentages of **(A)** total B220+ B-cells (****p < 0.0001), **(B)** T2 subset, and **(C)** MZ B-cells (**p = 0.002, *p = 0.022) in Mir146a^−/−^ (146a KO), Mir146a^−/−^ *TCR*β^−/−^ (DKO), and *TCR*β^−/−^ (TCRβ KO) mice. All non-annotated comparisons are not statistically significant (*n* = 4–7 mice/group, repeated in duplicate). **(D)** Representative FACS plots showing the MZ fraction in spleen in three mice groups.

Given that T cells show profound abnormalities in the *Mir146a*^−/−^ mice, another possibility is that there is a T-dependent pathway that leads to increased numbers of FO cells and decreased numbers of MZ B-cells. To test this, we bred mice deficient in αβ T cell receptor (*TCR*β^−/−^) onto *Mir146a*^−/−^ mice to make double knockouts *Mir146a*^−/−^, *TCR*β^−/−^ (DKO), and examined splenic subsets. As expected, *TCR*β^−/−^ and DKO showed higher number of B220+ cells (Figure 6A), given the deficiency of T cells skewing the immune populations. Hence, to compare between genotypes, we examined the percentage of B220+ cells for all given splenic subsets (i.e., T2, MZ shown). The T2 subset was similar in KO and DKO mice (Figure 6B), and we noted no differences in MZ subsets between *Mir146a*^−/−^ and DKO, indicating that this defect was not T cell dependent (Figures 6C,D). Furthermore, when comparing DKO with *TCR*β^−/−^ mice, DKO displayed statistically significant decreases in MZ cells, suggesting that the phenotype is indeed an effect of miR-146a in B-cells (Figures 6C,D). Taken together, our results indicate MZ deficit seen in miR-146a-deficient mice is likely B-cell intrinsic and is not dependent on the presence of hyperactivated T cells.

## Discussion

Here, we found that miR-146a deficiency has an effect on the differentiation of B-cells in the spleen. miR-146a is expressed more highly in MZ B-cells than in any other splenic B-cell subset, suggesting an important role in these cells. Indeed, the MZ B-cell subset in *Mir146a^−/−^* mice is markedly reduced. The T1 and T2 subsets that developmentally preceded MZ B-cells are higher in the spleen. Peripheral blood and lymphoid MZ B-cells are similar, suggesting that MZ splenic retention is intact. Furthermore, differentiation into plasmablasts is similar between KO and WT, and alterations in proliferation and apoptosis do not account for the MZ B-cell defect. Hence, we felt that this MZ B-cell abnormality arises from defective development of this subset within the spleen. Exploring the molecular mechanism of this block, we found a reduction in intracellular *Notch2* and its downstream transcriptional targets, implicating miR-146a-mediated regulation of a pathway required for the development of MZ B-cells. In identifying this pathway, we utilized a novel method of analysis, where we compared developmental transition-related transcriptomic changes between WT and KO mice. This was productive, as it highlighted many more genes that were potentially important than a simple cross-comparison between WT and *Mir146a*^−/−^ cells at a particular developmental change.

Because miR-146a is an important regulator of NFκB signaling, we wanted to understand whether the MZ B-cell phenotype that we observed is a consequence of hyperactivated NFκB. In myeloid and classic CD4+ and CD8+ T cells, miR-146a is induced by NFκB, and in turn targets the upstream adapter proteins Traf6 and Irak1, to cause negative feedback regulation of the NFκB response (8, 11). Thus, an overall increase in proinflammatory NFκB, as a consequence of loss of negative feedback, is responsible for many of the phenotypes seen in *Mir146a*^−/−^ mice. Knocking out the classical NFkB p50 in these mice resulted in a rescue of the myeloproliferative phenotype, but did not completely rescue the autoimmune pathology (12) and only partially corrected hyperactivation of T cells (11). We did not find telltale increases in key NFκB transcriptional targets at the RNA level and did not observe changes in Irak1 or Traf6 protein. However, Notch2 was clearly affected. NFκB and Notch2 appear to cooperate in MZ B-cell development as demonstrated by heterozygous knockout Notch2^+/−^ p50^+/−^ mice which show loss of MZ B-cells without affecting the FO B-cell population (25). Interestingly, non-canonical NFκB pathway *via* Relb was also shown to be important for development of MZ B-cells (26, 27). In our data set, NFκB response genes generally are downregulated at the T2-MZ transition, which would be consistent with miR-146a upregulation at that transition. However, we did not see direct evidence of these response genes or of known miR-146a targets (*Traf6 and Irak1*) being dysregulated in *Mir146a*^−/−^ mice. This would indicate that the main thrust of miR-146a activity is not focused on this pathway in MZ B-cells. miR-146a likely targets different pathways in different immune cells/tissues. Indeed, it was shown that in T regulatory cells (Tregs), miR-146a is highly expressed and likely targets *Stat1* in the interferon gamma pathway (IFNγ) (13). Our own recent work demonstrated *Egr1* to be a novel target of miR-146a in Myc-driven B-cell tumors (14). Hence, a multiplicity of targets for miR-146a exists in different cell types and under different contexts.

Our data demonstrate that Numb is a possible novel target of miR-146a in B-cells. Numb is increased in miR-146a-deficient T2 cells, which immediately developmentally precede MZ cells. Although Numb is a known regulatory of Notch, the reciprocal relationship between Numb and Notch2 is not seen in the same cells. Hence, the connection between miR-146a, Numb, and Notch2 cannot be definitively made. Given our findings, we speculate that a combination of the cellular kinetics of differentiation, balance between production and degradation of intracellular Notch2, and asymmetric cell division mediated by Numb (24) may underlie the relationships that we have observed. Indeed, the rates of production and degradation of intracellular Notch2 may be dramatically different once a cell has committed to the MZ B-cell fate. However, teasing out the nature of these relationships will take additional work and effort.

We also found that the MZ phenotype is T cell independent, supporting a B-cell intrinsic mechanism for the MZ defect in *Mir146a*^−/−^ mice. Other investigators have also endorsed the idea that MZ B-cell development is largely T-independent (2). In addition, the conditional knockout of Dicer in B-cells specifically led to an increase in MZ B-cells and decreased FO B-cells, suggesting that miRNAs can drive B-cell intrinsic phenotypes independent of T cells (6).

Our report demonstrates a specific B-cell subset abnormality in *Mir146a*^−/−^ mice, and we have identified a likely novel target in the MZ B-cell lineage. Our future studies will focus on how *Mir146a*^−/−^ B-cells contribute to the autoimmune phenotype, manifested by end-organ inflammation and production of anti-double-stranded DNA antibodies (8) characteristic of murine and human systemic lupus erythematosus (SLE). As the function of MZ B-cells in responses to specific antigen types has been postulated, it will be important to determine how this reduction in MZ B-cells, and the corresponding increase in FO cells, have functional consequences in tilting the balance toward clinical autoimmunity. Interestingly, miR-146a is expressed at low levels in peripheral blood mononuclear cells (PBMC) from patients with SLE (28) and treatment with chemically modified miR-146a agonists improved pulmonary hemorrhage in a pristane-induced lupus mouse model (29). Hence, it will be highly interesting to determine how miR-146a deficiency in B-cells contributes to autoimmune pathogenesis in murine models and ultimately in human disease.

## Ethics Statement

All mouse studies were approved by the UCLA Chancellor’s Animal Research Committee (ARC), the local IACUC body for UCLA.

## Author Contributions

JK designed studies, acquired data, performed analysis, and wrote the manuscript. NU and MP acquired data, performed analysis, and contributed to writing of manuscript. JC, MA, and TF performed data acquisition and analysis. KZ and MP performed data analysis. DR designed studies, performed interpretation and analysis, and wrote the manuscript.

## Conflict of Interest Statement

The authors declare that the research was conducted in the absence of any commercial or financial relationships that could be construed as a potential conflict of interest.
