# Supplementary material for: Regulation of Marginal Zone B-Cell Differentiation by MicroRNA-146a
Source: Front Immunol. 2017 Jan 16;7:670. doi: 10.3389/fimmu.2016.00670 (PMC5237642; doi:10.3389/fimmu.2016.00670)
Supplement: Supplementary file 1 [file presentation_1.pdf]

Supplementary Material

Regulation of marginal zone B-cell differentiation by microRNA-146a

Jennifer K King[1,2], Nolan M Ung[3], May H Paing[3], Jorge R Contreras[2,3], Michael O Alberti[3], Thilini R Fernando[3], Kelvin Zhang[4,5], Matteo Pellegrini [6,7,8], and Dinesh S Rao[3,7,8]

[1]Division of Rheumatology, Department of Medicine, UCLA STAR
[2]Cellular and Molecular Pathology Ph.D. Program, UCLA
[3]Department of Pathology and Laboratory Medicine, UCLA
[4]Department of Biological Chemistry, UCLA
[5]Howard Hughes Medical Institute, UCLA
[6]Jonsson Comprehensive Cancer Center, UCLA
[7]Molecular Cell and Developmental Biology, UCLA
[8]Eli and Edythe Broad Center of Regenerative Medicine and Stem Cell Research, UCLA

***Correspondence:**
Dinesh S. Rao, M.D., Ph.D.,
Assistant Professor
Department of Pathology and Laboratory Medicine
David Geffen School of Medicine at UCLA
650 Charles E Young Drive, 12-272 Factor
Los Angeles, CA 90095
Tel. 310-825-1675
Fax. 310-825-0814
Email drao@mednet.ucla.edu

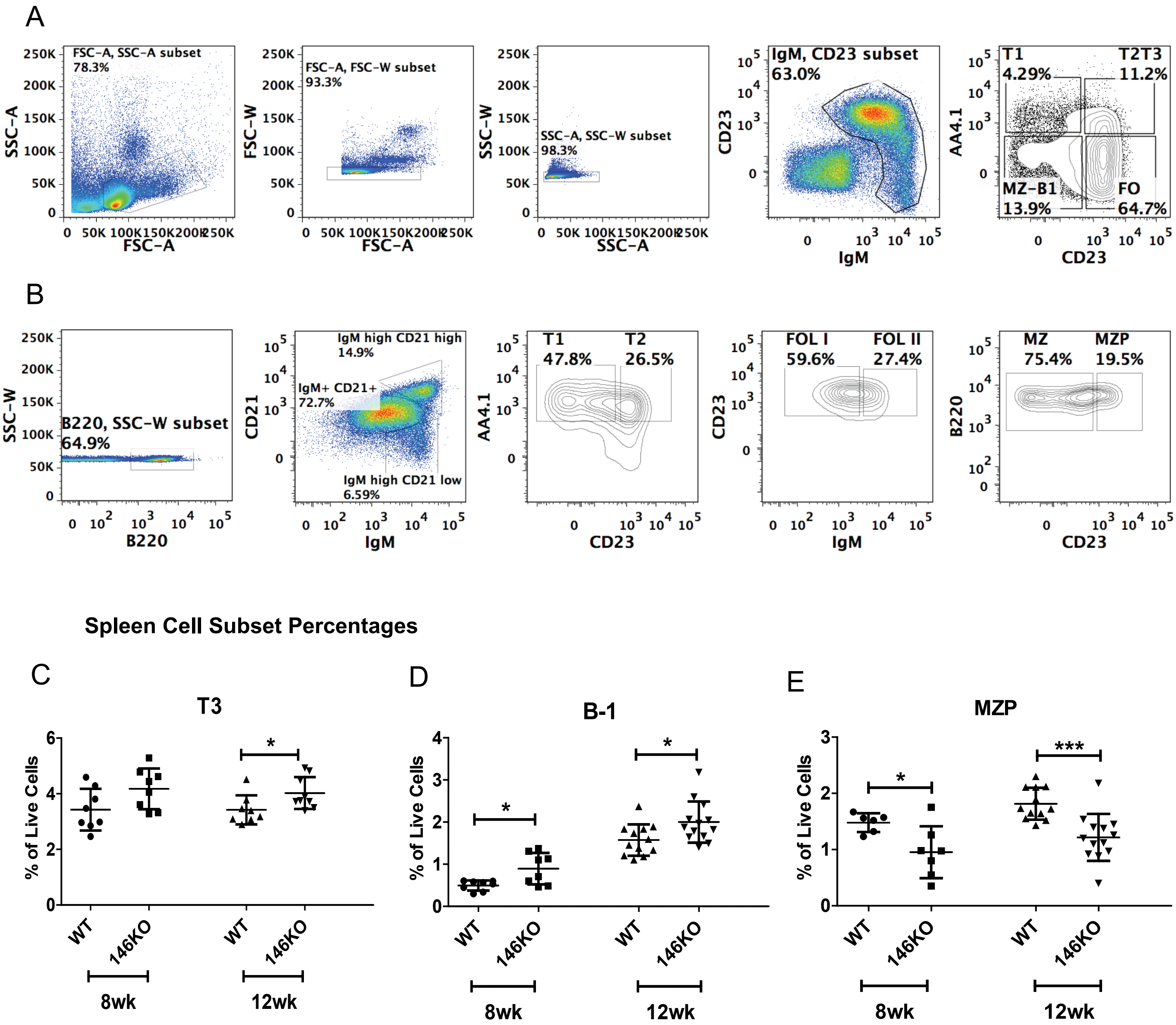

Supplement 1: *Mir146a*$^{-/-}$ mice exhibit increasing level of transitional precursors and B1 cells at a young age, but decreased marginal zone precursor cells. (A) Initial pre-gating FACS plots for spleen are shown. (B) From the SSC-A, SSC-W subset, an alternative gating strategy is shown to evaluate marginal zone precursor (MZP) cells. (C) Spleen transitional cell subset 3 (T3) show trend in 8 week *Mir146a*$^{-/-}$ knockout (KO) mice, and increased percentage of T3 cells at 12 weeks (*p=0.039; n=8-9 mice/group). (D) Splenic B-1 cells are increased in KO mice at 8 weeks (*p=0.012) and 12 weeks (*p=0.022). n= 8-13 mice/group. (E) Splenic MZP cells are decreased in KO mice at 8 weeks (*p=0.0234) and 12 weeks (***p=0.0004; n= 6-13 mice/group). All values are mean + SD.

Spleen Subset Cell Numbers

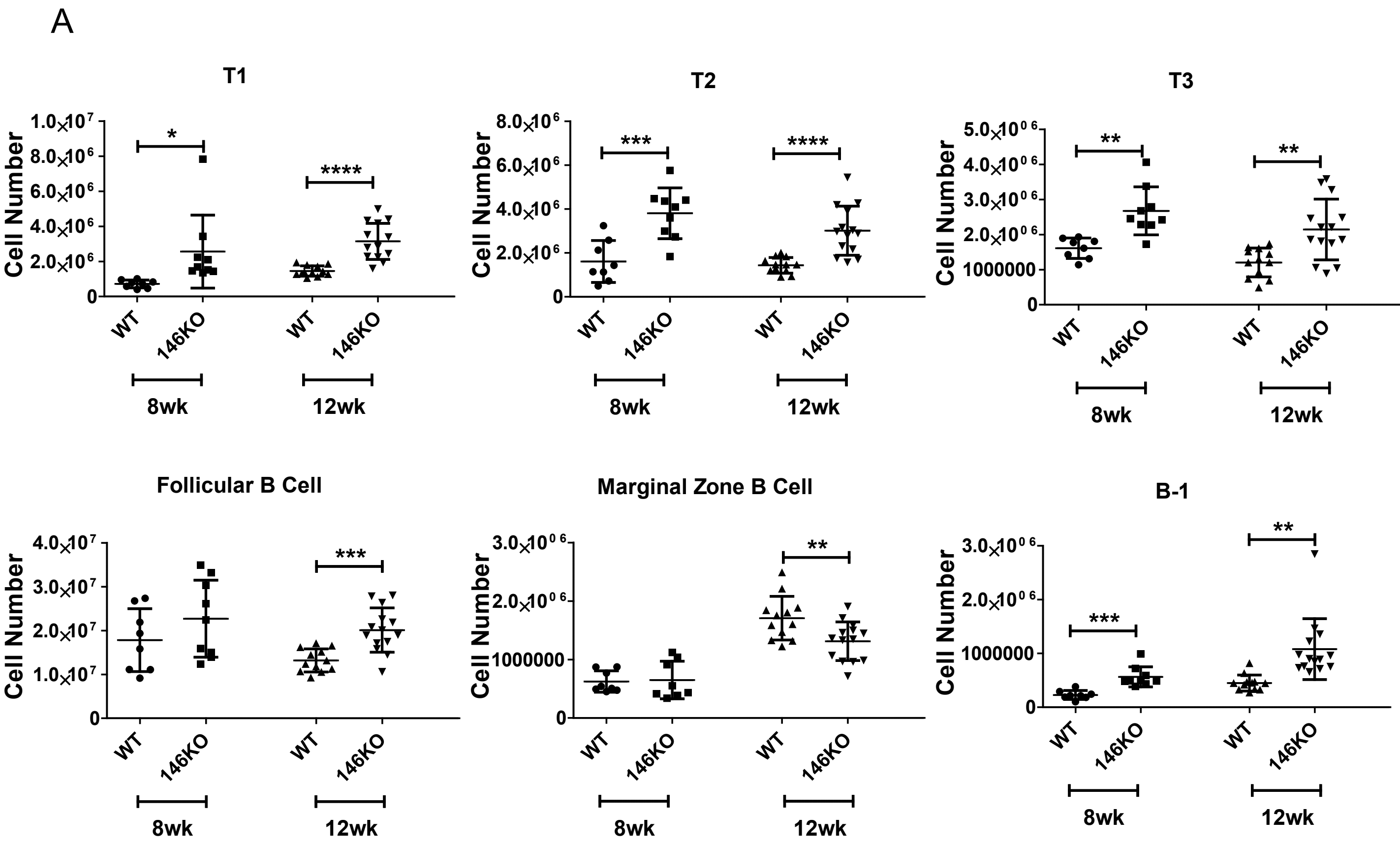

Supplement 2: *Mir146a*$^{-/-}$ mice have increased cell numbers in all splenic subsets, except marginal zone B cells at a young age. (A) Cell numbers of splenic subsets in 8 week (n=8 mice/group) and 12 week mice (n=12-13 mice/group) respectively are increasing in T1 (*p=0.0253, ****p<0.0001), T2 (***p=0.0007, ****p<0.0001), T3 (**p=0.001, **p=0.002), follicular (***p=0.0003), B1 (***p=0.0003, **p=0.001) cells. In contrast, marginal zone B cells are similar in 8 week-old mice or decreased in 12 week old mice (**p=0.0087) in spite of overall B cell increase in spleen. All values are mean + SD.

Spleen Subset Percentages in Aging mice

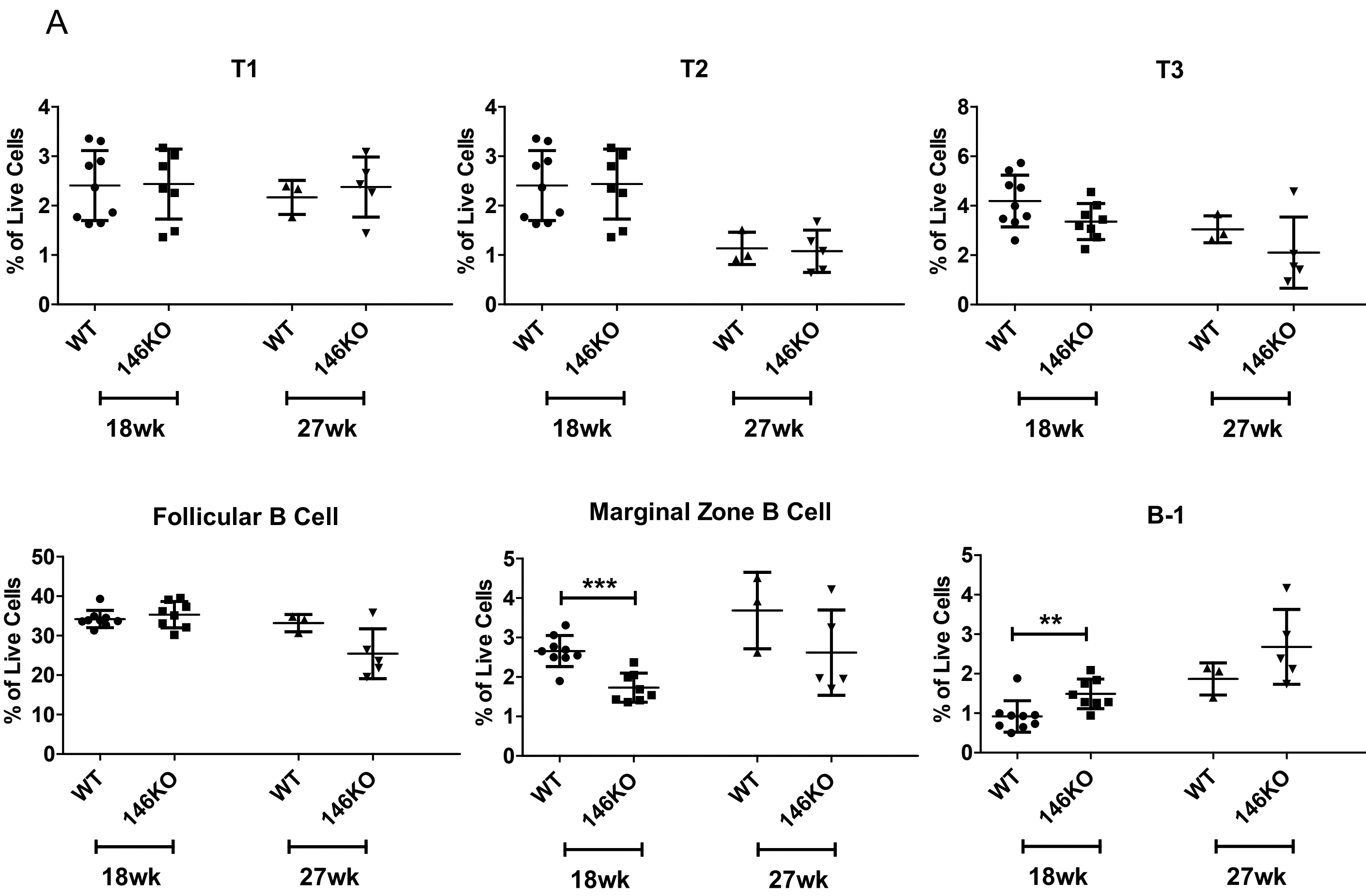

Supplement 3: Age-related *Mir146a*$^{-/-}$ mice inflammatory phenotype leads to decline in splenic B cells compartment. (A) Spleen subsets are shown in mice 18 weeks (n= 8-9 mice/group) and 27 weeks (n=3-5 mice/group) old showing normalization of percentages in T1, T2, T3 and follicular cells. By 27 weeks, the B-1 compartment is also similar between KO and WT. Marginal zone B cells continue to be decreased in KO mice compared to WT up through 18 weeks (***p=0.0002). However by 27 weeks, all B cell subsets show similar percentages in the spleen. All values are mean + SD.

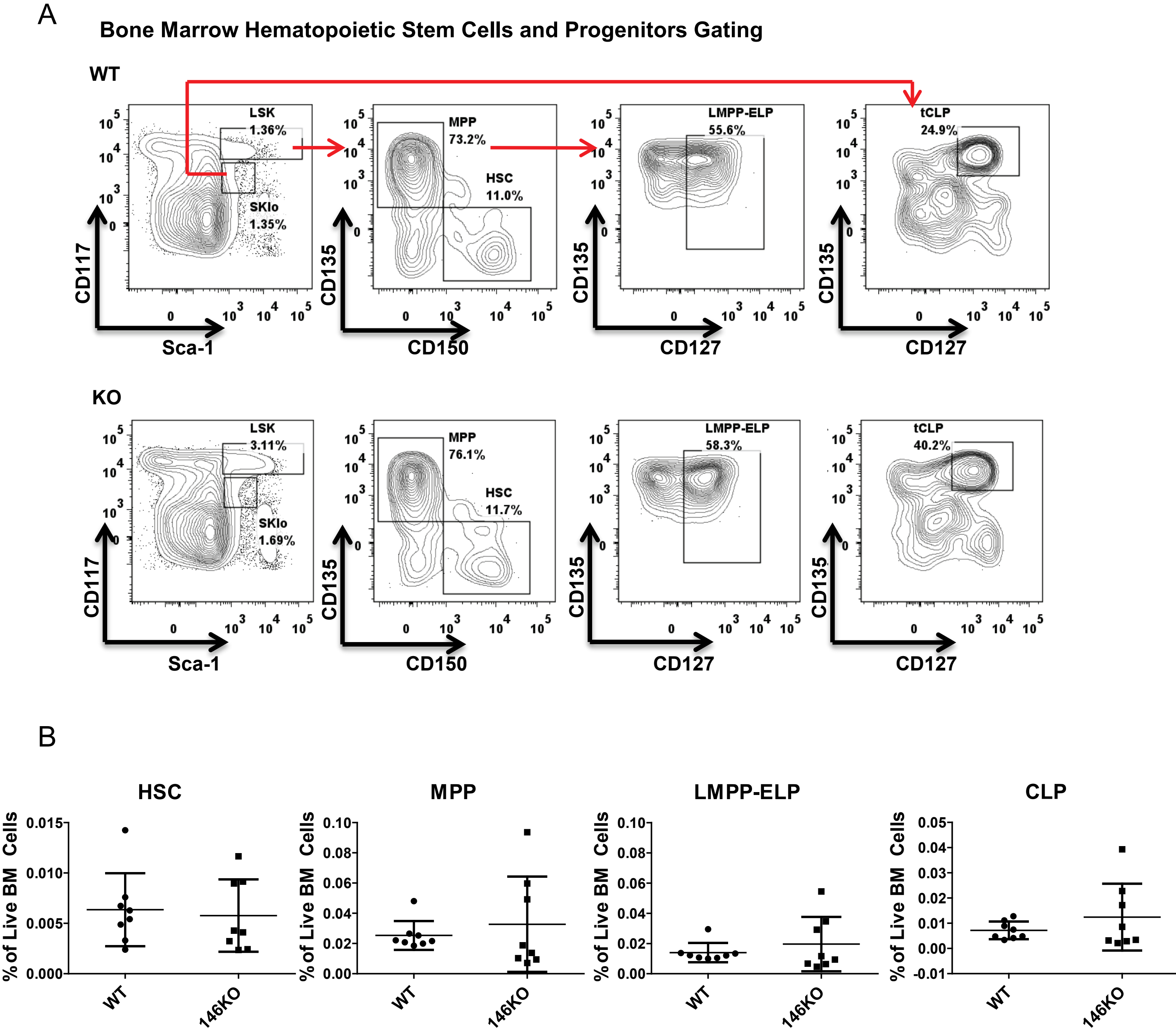

Supplement 4: *Mir146a*$^{-/-}$ mice show no differences in hematopoietic stem cell and stem cell progenitor cells at a young age. (A) Representative FACS plots of hematopoietic stem cells (HSC), multipotent progenitors (MPP), lymphoid-primed multipotent progenitors (LMPP), early lymphocyte progenitors (ELP), and common lymphoid progenitor (CLP). (B) Percentages of live cells of HSC and progenitor cells shown are not different between WT and KO mice at 8 weeks of age. All values are mean + SD.

A

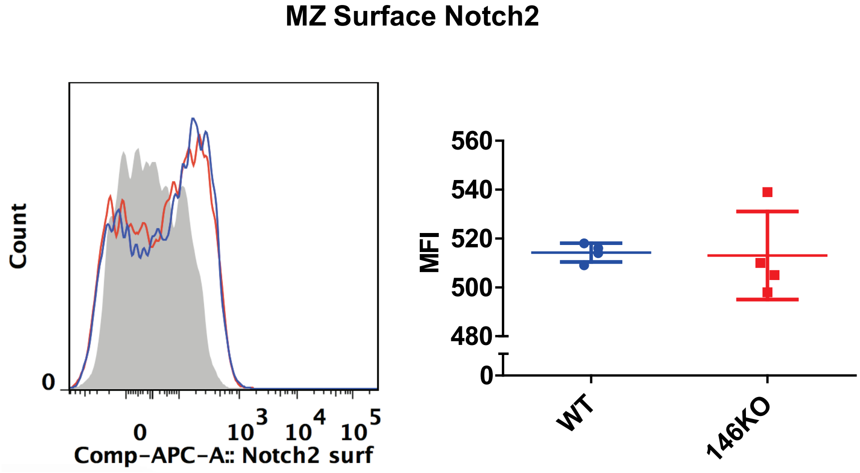

Supplement 5: *Mir146a$^{-/-}$* and WT mice have similar levels of surface Notch2 expression. (A) Representative FACS overlay of KO vs. WT surface Notch2 expression (left panel). Quantification of MFI surface Notch2 (right panel) show no difference (n=4mice/group).

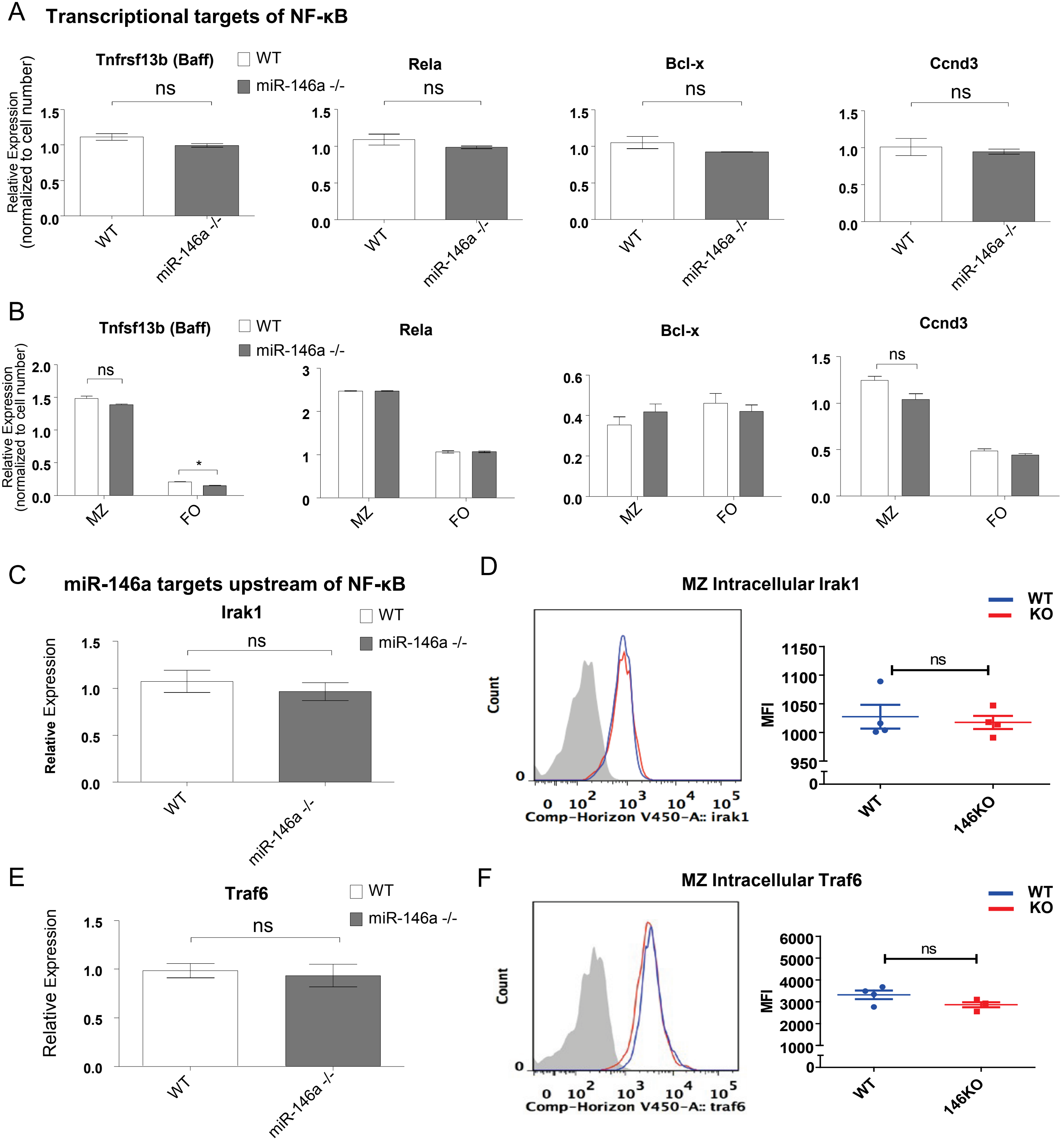

Supplement 6: Marginal zone B cell defect is not due to a lack of NF-κB. (A) Representative RT-qPCR analysis of NF-kB associated genes in bulk splenic B cells 72hrs after LPS stimulation. Repeated in duplicate. (ns = not significant) (B) Quantified relative expression of NF-κB associated genes in splenic B cell subsets using RT-qPCR (FO *p=0.02; repeated in duplicate). (C) RT-qPCR analysis of Irak1 in bulk splenic B cells 72hrs after LPS stimulation from WT and KO mice (repeated in duplicate). (D) FACS of MZ B cells as an overlapping histogram (left panel) and quantitation of median fluorescence intensity (MFI) (right panel) of intracellular staining of Irak1 (n=4 mice/group). (E) RT-qPCR analysis of Traf6 expression in bulk splenic B cells 72hrs after LPS stimulation from WT and KO mice (repeated in duplicate) (F) FACS of MZ B cells as an overlapping histogram (left panel) and quantitation of median fluorescence intensity (MFI) (right panel) of intracellular staining of Traf6 (n=4 mice per group). All values are mean + SEM.

Supplementary Table 1: A list of antibody stains for FACS analysis.

Bone Marrow B Progenitor Cell Subsets		
**Antigen**	**Fluorochrome**	**Company**
B220 (RA3-6B2)	PerCP-Cy5.5	BioLegend
IgM	PE	SouthernBiotech
CD43 (S7)	APC	BD Biosciences
CD24 (M1/69)	PECy7	eBioscience
IgD (11-26c.2a)	APC-Cy7	BioLegend
Ly51 (6C3)	Biotin	BioLegend
Streptavidin	APC-eFluor 780	eBioscience
**Spleen Subsets**		
**Antigen**	**Fluorochrome**	**Company**
B220 (RA3-6B2)	PerCP-Cy5.5	BioLegend
IgM	PE	SouthernBiotech
CD21 (7E9)	FITC	BioLegend
CD23 (B3B4)	PECy7	BioLegend
CD93 (AA4.1)	APC	eBioscience
**Bone Marrow Hematopoietic Stem Cell and Progenitor Cells**		
**Antigen**	**Fluorochrome**	**Company**
CD150 (TC15-12F12.2)	PE	BioLegend
Sca1 (D7)	PerCP-Cy5.5	BioLegend
CD127 (A7R34)	PE-Cy7	BioLegend
CD135 (A2F10)	APC	eBioscience
CD117 (2 B8)	APC-Cy7	BioLegend
CD8a (53-6.7), B220 (RA3-6B2), TCR β (H57-597) , TCR γ/δ (GL3), NK1.1 (PK136), Gr1 (RB6-8C5), Ter119 (TER-119)	Biotin	BioLegend
CD4 (GK1.5), IgM (II/41)	Biotin	eBioscience
Streptavidin	eFluor 450	eBioscience
**Intracellular Staining**		
**Antigen**	**Fluorochrome**	**Company**
Notch2 (HMN2-35)	APC	BioLegend
Numb (polyclonal)	APC	Novus
IRAK1 (D51F7)		Cell Signaling
TRAF6 (EP591Y)		Abcam
IgG (Poly4064)	Brilliant Violet	BioLegend

Supplementary Table 2: Hematopoietic Cell Subset Definitions

**Bone Marrow B Progenitor Cell Subsets**	**Surface Marker Identification**
A	$B220^{+}$ $CD43^{+}$ $CD24^{-}$ $Ly51^{-}$
B	$B220^{+}$ $CD43^{+}$ $CD24^{+}$ $Ly51^{-}$
C	$B220^{+}$ $CD43^{+}$ $CD24^{+}$ $Ly51^{+}$
D	$B220^{+}$ $CD43^{-}$ IgM^{-}
E	$B220^{+}$ $CD43^{-}$ IgM^{+}
F	$B220^{++}$ $CD43^{-}$ IgM^{+} IgD^{+}
**Spleen subsets**	**Surface Marker Identification**
T1	$CD23^{-}$ $AA4^{+}$ IgM^{high} $CD21^{-/low}$
T2	$CD23^{+}$ $AA4^{+}$ IgM^{high} $CD21^{low}$
T3	$CD23^{+}$ $AA4^{+}$ IgM^{low} $CD21^{low}$
Follicular B cell	$CD23^{+}$ $AA4^{-}$ IgM^{low} $CD21^{int.}$
Marginal Zone B cell	$CD23^{-}$ $AA4^{-}$ IgM^{high} $CD21^{high}$
Marginal Zone B cell Precursor	$CD23^{+}$ $AA4^{-/low}$ IgM^{high} $CD21^{high}$
**Bone Marrow Hematopoietic Stem Cell and Progenitor Cells**	**Surface Marker Identification**
Hematopoietic Stem Cell	Lin^{-} $CD117^{high}$ $Sca1^{high}$ $CD150^{+}$
Multipotent Progenitor	Lin^{-} $CD117^{+}$ $Sca1^{+}$ $CD135^{+}$
Lymphoid-Primed Multipotent Progenitor	Lin^{-} $CD117^{high}$ $Sca1^{high}$ $CD135^{+}$ $CD127^{-}$
Common Lymphoid Progenitor	Lin^{-} $CD117^{low}$ $Sca1^{low}$ $CD135^{+}$ $CD127^{+}$

Supplementary Table 3: A list of the primers used in the RT-qPCR analysis.

q-m-Ccnd3-F	CGAGCCTCCTACTTCCAGTG
q-m-Ccnd3-R	GGACAGGTAGCGATCCAGGT
q-m-bclx-F	GACAAGGAGATGCAGGTATTGG
q-m-bclx-R	TCCCGTAGAGATCCACAAAAGT
q-m-Tnfrsf13b-F	ATGGCATTCTGCCCCAAAGAT
q-m-Tnfrsf13b-R	ATGGTCGTAGTACCTGCCTTG
q-m-NFkB-F	ATGGCAGACGATGATCCCTAC
q-m-NFkB-R	TGTTGACAGTGGTATTTCTGGTG
q-m-RelA-F	AGGCTTCTGGGCCTTATGTG
q-m-RelA-R	TGCTTCTCTCGCCAGGAATAC
q-m-Irak1-F	ACTCCAGAGAAGTCCCAACCA
q-m-Irak1-R	CAGGAATGCAGGGTAGCAGAG
q-m-Irf2-F	AATTCCAATACGATACCAGGGCT
q-m-Dtx3-F	CCCAGCTACGAGAAGTATGGC
q-m-Dtx3-R	TCAAATGCCTTTCGGAACAGG
q-m-Dtx1-F	TACATGCAGAAGGTGAAAAACCC
q-m-Dtx1-R	CGCCCTCATAGCCAGATGC
q-m-Dtx4-F	CGCCCAGTTCGACGAAACTA
q-m-Dtx4-R	TGATGCCGACTTCCATGTCATA
q-m-Tnfrsf13b-F	ATGGCATTCTGCCCCAAAGAT
q-m-Tnfrsf13b-R	ATGGTCGTAGTACCTGCCTTG
q-m-Notch2-F	GACTGCCAATACTCCACCTCT
q-m-Notch2-R	CCATTTTCGCAGGGATGAGAT
q-m-Hes5-F2	ATGGCCCCAAGTACCGTGGCG
q-m-Hes5-R2	AGCTTGGAGTTGGGCTGGTG
q-m-Hes1-F	CCAGCCAGTGTCAACACGA
q-m-Hes1-R	AATGCCGGGAGCTATCTTTCT
q-m-Traf6-F1	GCACAAGTGCCCAGTTGAC
q- m-Traf6-R1	TGCAAAATTGTCGGGAAACAGT
